# Effect of Frugality and Cognition on Forest Health Tourism Intention–A Mediating Effect Analysis Based on Multigroup Comparison

**DOI:** 10.3389/fpsyg.2022.844628

**Published:** 2022-04-29

**Authors:** Ying Li, Qiang Han, Ting Wen

**Affiliations:** Business School, Liaoning University, Shenyang, China

**Keywords:** frugality, cognition, mental account, forest health tourism, behavioral intention

## Abstract

At present, the market demand for forest health tourism is weak. The main purpose of this study is to investigate whether frugality inhibits the intention of forest health tourism and whether the positive effect of cognition on the intention of forest health tourism can compensate for the inhibition of frugality. Based on mental account theory and planned behavior theory, this study constructs a structural equation model with intermediary variables—health consumption mental account and forest health consumption attitude. According to the results of the path analysis of the data, which was collected through the questionnaire survey of urban residents, the positive influence of cognition can compensate for the inhibitory effect of frugality. On this basis, mediating effect analysis based on multigroup comparison is further carried out. This study verifies for the first time the inhibitory effect of frugality on the intention for forest health tourism, enriches the theoretical system of tourism consumer behavior, and provides a scientific basis for the market positioning of forest health and the formulation of marketing strategies.

## Introduction

At present, with the increase in the subhealthy population and the acceleration of population aging, the number of chronic diseases in China is growing rapidly (Li et al., 2019). According to existing statistics, the disease burden caused by chronic diseases in China exceeds 70%^1^ of the total burden of disease. With the implementation of the Healthy China Strategy and increasing public health awareness, the forest health industry came into being. The forest health and maintenance industry is a kind of tourism service complex that relies on forest resources (forest parks) and integrates the concepts of tourism, leisure, health preservation, sports, medical treatment, and poverty alleviation into the development of forest ecological services. It has become a new form of integrated development of ecological forestry, tourism, and health services. Forest health tourism comprise various vacation tourism activities that are beneficial to human physical and mental health based on high-quality forest resources (Deng, 2016). Studies have shown that the large number of negative oxygen ions and phytochemicals in forest environments play an important role in the prevention and adjuvant treatment of mental and chronic non-communicable diseases (Lee et al., 2015; Ohe et al., 2017). The development of the forest health tourism industry plays an important role in improving people’s well-being, protecting forest resources, and promoting the transformation and upgrading of the forest economy (Cong and Zhang, 2016).

Since 2015, China has vigorously promoted the forest health industry (Cong and Zhang, 2016; Chen, 2021). Although thousands of units have been rated national forest health base pilot construction units, only 96 units have been officially awarded the national forest health base title. Due to the sluggish market demand for forest health tourism, these units have made little investment in forest health projects. The main features include forest trails and forest yoga, with Taiji and other activities held occasionally. Most studies on forest health are limited to the concept of forest health, problem analysis, and countermeasures (Sun et al., 2021). Previous studies on forest health tourism intention using the theory of planned behavior (including the extended theory of planned behavior) (Xie et al., 2019) did not provide specific information on the effect of tourism destinations and prices on consumer attitudes, which made it difficult to measure real consumer attitudes and specific behavioral intentions. At present, there is few literature exploring the effect of frugality and cognitive levels on forest health tourism intention. In previous studies on sociology and management, the research on frugality has not attracted enough attention from scholars (Mark, 2016). There are relatively few research results on frugality, and frugality, as an important factor affecting consumers’ willingness for forest health tourism, has been ignored by scholars. In addition, there has not been a clear definition and academic definition of forest health tourism cognition.

As the core concepts in the field of psychological research, the research on mental account and theory of planned behavior has been very mature, but they have never been used as mediator variables in the research on forest health tourism intention. Mental account theory tells that people’s decision-making depends on mental accounts rather than real accounts, that is, people unconsciously allocate wealth to different accounts for management, and different mental accounts have different accounting methods and psychological operation rules. The way and rules of mental accounting are different from those of economics and mathematics, and often affect individual decisions in unexpected ways (Liu P. et al., 2019). In the field of human behavior prediction, the theory of planned behavior is one of the most commonly used explanatory models (Ajzen, 2011). This theory points out that the intention of attitudes, subject norms, and perceptual behavioral control together shapes individual behavioral intentions and behaviors. The combination of mental account theory and theory of planned behavior will establish a possible interpretation framework for forest health tourism intention.

To sum up, the current research on forest health tourism at home and abroad is mostly limited to the development status and concept connotation of forest health tourism, and the research and discussions are not in-depth, and there are few studies on the influencing factors of forest health tourism intention. At present, forest health tourism has attracted much attention, but the forest health tourism market is weak. Therefore, focusing on understanding the factors that hinder consumers’ forest health tourism willingness, to explore the strategy of driving consumers’ forest health tourism willingness will be the focus of future forest health tourism research. This study explores the role of frugality and cognition in forest health tourism, which not only fills the gap of research on factors influencing forest health tourism intention, but also provides corresponding countermeasures and suggestions for the development of forest health tourism destination. At the same time, health account and consumer attitude are taken as mediating variables in this study, which is another application of psychological concepts in the field of consumer behavior.

Based on mental account theory and planned behavior theory, this study constructs an analytical framework to reveal the comprehensive influence of mental account, attitude, and subjective norms on forest health tourism intention from the perspective of frugality and cognition and uses a multigroup analysis method to explore the differences between groups, aiming to provide a decision-making reference for forest health tourism enterprises to accurately determine product market and formulate marketing strategies to promote the economic development of forest areas.

## Theoretical Review

### Frugality Habits

As a core concept of traditional consumption in China, frugality has gradually been incorporated into consumption ethics (Tan et al., 2015). Frugality is the conscious reduction of consumption according to one’s intentions and defines an anti-consumption lifestyle (Albinsson et al., 2010; Nepomuceno and Laroche, 2015; Kropfeld et al., 2018; Khamis, 2019). Frugal consumers are careful in their daily product purchases and consumption habits (Rose et al., 2010), resulting in a negative effect of frugality on consumption expenditure (Pan et al., 2019), which means they reduce their consumption intentions (Philp and Nepomuceno, 2019). Frugality is generally considered to involve the planned use of resources and avoidance of wasteful behavior (Young, 1986), which does not fail to meet people’s basic life needs but does require people to restrain their desires (Gao, 2010). Consumer ethics holds that frugality and luxury are two opposite moral evaluation criteria (Xing, 2018) and regards frugality as morally positive (Muradian, 2019).

At present, frugality is divided into two types from the perspective of behavioral motivation. The first is extremely purposeful frugality, which involves achieving a more important long-term goal by limiting short-term consumption impulses and allocating only resources (Lastovicka et al., 1999). For example, people use the money saved in daily life to purchase luxury goods and real estate. The second is simple frugality, which is frugality behavior formed only to save resources and not used to achieve a specific consumption goal (Pepper et al., 2009). The second type of frugality, the habit of frugality consumption formed to conserve resources (money, hydropower, etc.), is considered in this study.

### Mental Account Theory

Under the assumption of complete consumer rationality, traditional consumption expenditure theory cannot perfectly explain the actual thinking modes, and consumption decisions of consumers (Ge et al., 2019). Individual consumption decisions are affected by mental accounts (Liu P. et al., 2019). Mental accounting is the mental cognitive process by which individuals or families classify, store, encode and estimate the income sources and payment directions of wealth mentally when making a consumption decision (Kivetz, 1999; Li and Ling, 2007) and involves evaluating a variety of choices individually (Kahneman and Tversky, 1984). Since Thaler (1999) formally proposed mental account theory, “Mental Account” has been summarized as a cognitive operational stereotype used by individuals and families to record, organize, and evaluate consumer activities (Benartzi and Thaler, 1999).

In recent years, domestic scholars have systematically conducted research on mental accounts and consumption abroad (Liu Y. et al., 2019) and explored the mechanism of tourism consumption decision-making based on mental accounts (Wang, 2019). The health account in this study is primarily the mental account established by urban residents for health consumption expenditures (fitness, health products, health holidays, etc.).

### Theory of Planned Behavior

The theory of planned behavior was developed after introducing the concept of “perceived behavioral control” on the basis of the theory of reasoned action (Ajzen, 1991), which provides a systematic analytical framework for explaining and predicting individual behavior research (Yan et al., 2019). According to the theory of reasoned action, most of people’s behaviors are under their own control and rational, determined by volitional factors, i.e., behavioral attitudes and subjective norms, without considering the situation that behaviors are not under individual control (Han, 2015). In order to enhance the explanatory power of theory of reasoned action, Ajzen (1991) introduced the variable of perceived behavior control as the third variable influencing behavior along with behavioral attitude and subjective norms, and pointed out that individual behavior could be directly determined by the perceived behavior control and behavior willing variables, thus formed the theory of planned behavior (Qiu, 2017). According to the theory of planned behavior, planned behavioral intention is affected primarily by three factors: behavioral attitude, subjective norms, and perceived behavioral control (Yang and Zhu, 2018). Behavioral attitude is an individual’s preference or attraction evaluation of a particular behavior (Juschten et al., 2019). Subjective norms are the social pressure individuals experience when deciding whether to engage in a particular action (Lin and Li, 2016). Perceived behavioral control is the degree of ease or difficulty individuals feel when performing a particular behavior (Goh et al., 2017). Through empirical research, many scholars have proven that this theory has strong universality and practical significance for predicting various planned behaviors of human beings in actual environments (Xu et al., 2016).

As the theory of planned behavior applies to the prediction of a specific behavior, this study provides specific information on forest health destinations (130–160 km from the place of residence), forest environment pictures, time (at least one night), accommodation prices, and forest health curriculum costs that affect participation intention in the questionnaire. There are three types of accommodations available: camping with tents (no accommodation), cabins with outdoor bathrooms ($84 per night, with breakfast), and star hotel rooms ($200 per night, with breakfast). Accommodations include a nutritious lunch and supper. The total cost per capita is between 60 and 90 yuan. Forest rehabilitation courses include three types: free self-help, 20–60-yuan large-scale professional courses and 60–100-yuan professional small-scale courses. Most urban residents have both the time and money to participate in forest health tourism, which means that the influence of perceived behavioral control on urban residents can be ignored. Therefore, this study measures the influence of only urban residents’ behavioral attitudes and subjective norms on their forest health tourism intentions.

The “cognition-attitude-behavior” model can further explain the influence mechanism of cognition on attitude and expand the original theory of planned behavior. Cognitive psychology holds that people’s objective behavior is the external manifestation of their internal information cognitive processes and is the basis for whether an individual takes a specific action. Consumers’ preferences and behavioral intentions are directly or indirectly affected by their cognitions (Cooke and Sheeran, 2004). Under the influence of cognitive rationality, Western mainstream philosophy emphasizes the decisive role of rational cognition on attitudes (Liu, 2017), and behavioral attitudes directly affect individual behavioral intentions (Ajzen, 1991). Therefore, when explaining the role of cognition, the “cognition-attitude-behavior” theoretical model is gradually established; that is, when an individual perceives something, it produces a corresponding cognition, which affects the individual’s attitude toward the thing through cognition and then affects the individual’s behavioral intention (Deng et al., 2018). On the whole, the more information individuals have, the higher their degree of identity, and the more vulnerable they are to their attitudes toward the matter, thus affecting their behavioral intentions.

## Research Hypothesis

### Frugality, Health Account, and Behavioral Attitude

Since the Chinese population regards frugality as a traditional virtue, it informs the culture’s unique values in life consumption (Wu et al., 2012), which is obvious in the consumer mental account. Consumers divide their wealth into several separate accounts in daily life, and their mental accounts vary with commodities (Li et al., 2014). Consumers’ frugality habits will affect the size of health accounts, and even determines the existence of health accounts. Especially when forest health tourism exists as a kind of hedonic consumption, tourists with strong frugality habit will even consider closing the health account. In the process of exploring the influence of frugality on health accounts, this manuscript holds that the more frugal urban residents are, the smaller their health accounts may be, according to the mental account theory. Accordingly, the following assumption is made:

H1: Frugality has a negative effect on health accounts.

In previous frugality studies, frugality has always been regarded as a good social morality, emphasizing rational planning and enjoyment of life (Deng and Cheng, 2002), and has had many effects on individual psychology and attitudes. Frugality is aimed at individual desires and does not limit people’s normal life needs (Zhao and Gao, 2018). However, individual consumption attitudes are still more or less influenced by frugality. As a restraint of self-consumption, frugality has an effect on the consumer attitude of tourists to participate in forest health tourism from the level of consciousness (Li and Huang, 2014). In addition to the necessary life consumption expenditure, consumers’ consumption expenditure attitude will be constrained by frugality when facing hedonic consumption. Under normal circumstances, consumers affected by frugality consciousness will reduce their spending on hedonic consumption. Forest health tourism belongs to hedonic consumption, and tourists with strong frugality habit will be bound by certain constraints, thus affecting consumer attitudes toward forest health tourism. In the process of exploring the effect of frugality on the consumer attitude of tourists participating in forest health tourism, this manuscript holds that the higher the degree of frugality of tourists, the worse the attitude of tourists participating in forest health tourism. Accordingly, the following assumption is made:

H2: Frugality has a negative effect on forest health tourism attitudes.

### Cognition and Health Account, Behavioral Attitude

According to mental account theory, consumers’ consumption decisions depend on whether they have mental accounts for the corresponding commodities (Thaler, 2008). As mentioned above, frugal consumption habits of tourists affect the establishment of health accounts, but on the other hand, high-cognitive tourists can perceive greater psychological effects when they participate in forest health tourism consumption. The greater the psychological utility perceived by tourists, the more likely they are to set up a health account, thus improving their behavioral intention of forest health tourism consumption (Zeng et al., 2016). To sum up, this manuscript holds that urban residents with a higher awareness of the role of forest health will think that a vacation in a forest environment is a worthwhile consumption choice and are more likely to set up health accounts, resulting in different forest health tourism consumption intentions. Accordingly, the following assumption is made:

H3: Cognition has a positive effect on health accounts.

When consumers face consumption decisions, they start the process of income and expenditure assessment. Meanwhile, cognitive differences lead to differences in mental utility perception and then produce different consumer behaviors (Zeng et al., 2016). When evaluating the consumption expenditure and health benefits of forest health tourism, compared with urban residents with a low awareness of forest health effects, urban residents with a high awareness will perceive greater mental utility, resulting in positive consumer attitudes. At the same time, according to the “cognition-attitude-intention” theory, cognition will affect the attitude of tourists to participate in forest health tourism in the process of consumption decision-making evaluation (Deng et al., 2018). Therefore, this manuscript argues that the higher urban residents’ awareness of forest health is, the better their attitudes toward forest health tourism behavior. Accordingly, the following assumption is made:

H4: Cognition has a positive effect on attitude toward forest health tourism.

### Health Accounts, Subjective Norms, Behavioral Attitudes, and Forest Health Tourism Intentions

Forest health tourism intention (health intention in brief) is the action tendency of urban residents to participate in forest health tourism, which is closely related to the occurrence of the corresponding behavior. Although the study found that behavioral intention is the most important explanatory variable of the behavior (Godin and Kok, 1996), tourism decision-making, as a complex process full of uncertainty (Seow et al., 2017), is affected by many factors, including mental accounts. Therefore, mental account theory has been gradually introduced into consumer purchase decision-making research (Tian et al., 2016). Studies have shown that mental accounts affect consumers’ consumption intentions and behaviors (Li et al., 2012). The fund levels of mental accounts vary and cannot replaced each other. Forest health tourism consumption belongs to health consumption. Whether urban residents have a health account and the size of the health account directly determine the willingness of urban residents to participate in forest health tourism. Based on the effect of mental accounts on behavior willingness, this study suggests that the greater the number of health accounts owned by urban residents, the stronger their intentions to participate in forest health tourism. Accordingly, the following assumption is made:

H5: Health accounts has a positive effect on forest health tourism intention.

In general, individual behavior is affected by social pressure (Lv, 2019). That is, subjective norms directly affect individual behavioral intention (Ajzen, 1991). The effect of subjective norms on individual behavioral intention has been verified in many social behavior studies (Verma and Chandra, 2018; Wang et al., 2018). If individuals do not comply with a code of conduct and ignore the social pressure from their groups, they will be excluded by their groups (Lv, 2019). Therefore, this study claims that urban residents will ask their relatives and friends for their opinions and suggestions before forest health tourism. The more people around them support and advocate forest health tourism, the stronger their intention to participate. Accordingly, the following assumption is made:

H6: Subjective norms has a positive effect on forest health tourism intention.

Consumer attitude refers to an individual’s preference for a certain behavior. Ajzen (1991) predicts through theory of planned behavior that consumer attitude will directly affect an individual’s behavioral intention. Individuals’ intentions to participate in a behavior increase with their preferences for the behavior. Related studies have shown that individual behavioral attitudes directly affect behavioral intention (Kaushik et al., 2015; Ru et al., 2018). It can be speculated that when tourists have a positive consumer attitude toward forest health tourism, their behavioral intention to participate in forest health tourism will be stronger. This positive consumer attitude will not only affect tourists themselves, but also affect the relatives and friends around them. Therefore, behavioral attitudes reflect individuals’ preferences for forest health behavior. Urban residents with positive behavior attitudes are willing to participate in forest health tourism and invite others to join. Accordingly, the following assumption is made:

H7: Behavioral attitude has a positive effect on intention for forest health tourism.

Considering hypotheses H1–H7, hypotheses regarding the mediating effects of health accounts and behavioral attitudes are proposed:

H8a: Health accounts have a significant mediating effect between frugality and forest health tourism intention.
H8b: Health accounts have a significant mediating effect between cognition and forest health tourism intention.
H9a: Behavioral attitude plays a significant mediating role between frugality and forest health tourism intention.
H9b: Behavioral attitude plays a significant mediating role between cognition and forest health tourism intention.

**FIGURE 1:**
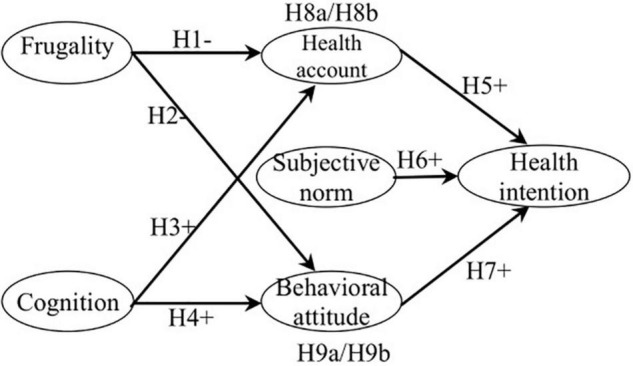
Theoretical model.

In summary, the theoretical model of this study is shown in Figure 1.

## Research Design

### Questionnaire Design and Variables Measurement

Although most of the variable measurement items in this study use scales that have been validated in China and abroad, a preliminary investigation of the questionnaire was still carried out to ensure the content validity of the questionnaire, and some items were modified and optimized according to research feedback. After expert review, the final questionnaire was determined (see Table 1).

**TABLE 1:** Measurement items.

Variable	Measurement items	References
Frugality	I prefer simple and clean economy hotels over more expensive business hotels.	Pepper et al., 2009; Wu et al., 2012; Hampson and McGoldrick, 2017
	When shopping, I always shop around, trying to use the least money, to achieve the maximum purpose.	
	I will save paper boxes, plastic bottles and other recyclable items and then sell them to recyclers.	
Cognition	Plant bacteriocins (Findol) and negative oxygen ions released from forests can promote human metabolism and improve immunity.	Lee et al., 2015; Ohe et al., 2017; Kleindl et al., 2018
	Forest environments can reduce the secretion of adrenaline to some extent and increase the secretion of dopamine in the eyes and improve vision.	
	Forest environments can reduce fatigue, promote relaxation, relieve stress and regulate mental health.	
	Sitting meditation or exercise in the forest can improve memory, hearing and sleep quality.	
	Forest environments play an important role in improving immunity and preventing epidemics.	
Behavioral attitude	I think forest health tourism is a symbol of life interest and quality of life that is worthy of consumption.	Xie et al., 2019
	I think forest health tourism is more effective than exercise in urban parks.	
	I think forest health tourism is worth more than buying health products at the same price.	
	I think forest health tourism is more worthwhile than participating in indoor club fitness at the same price.	
	Compared with not taking part in forest health tourism, I think taking part one time is beneficial for health.	
Subjective norm	My relatives’ participation in forest health tourism would affect me.	Deng, 2012; Xu et al., 2016; Ji and Nie, 2017
	My friend’s participation in forest health tourism would affect me.	
	Forest health tourism information from social media would affect me.	
Health account	The amount my family spends annually on health products; fitness (including swimming pool, fitness club and gym); and hot spring, forest and seaside vacations is.	Henderson and Peterson, 1992; Kivetz, 1999; Thaler, 1999
	The annual cost of health care products for my family is.	
	The annual spending range of my family for swimming pools, fitness clubs, stadiums and other places is.	
	The annual expenditure range of my family for forest health tourism consumption is.	
Health intention	I would like to advise others to participate in forest health tourism.	Assaker et al., 2011; Prayag et al., 2017; Xu and Li, 2018
	I would like to invite others to participate in forest health tourism.	
	I would like to join a Forest Health Tourism WeChat Communication Group.	

The questionnaire includes the following six parts:

1. Urban resident frugality measurement. This part uses the scale designed by Pepper et al. (2009) and Hampson and McGoldrick (2017), and draws on the three dimensions adopted by Wu et al. (2012), namely, the ratio of goods to goods, the amount of input and output, and the best use of goods. Items measure eating, clothing, and housing. After the reliability test and exploratory factor analysis, three items were finally retained.
2. The cognition measurement of urban residents on the role of forest health tourism is based on studies by Lee et al. (2015), Ohe et al. (2017), and Kleindl et al. (2018).
3. Urban residents’ attitudes toward forest health tourism and subjective norm measurement were surveyed using items measuring behavioral attitude adapted from the scale of Xie et al. (2019). On the basis of the original scale items, according to the needs of this study, the corresponding restrictions were added. The measurement items for subjective norms were taken from the scales designed by Deng (2012), Xu et al. (2016), and Ji and Nie (2017).
4. The health accounts of urban residents were measured based on the range of annual per capita spending on health products, fitness, travel, and vacation after the end of COVID-19 using nine options, ranging up to spending more than 5,000 yuan.
5. The items measuring urban residents’ forest health tourism intentions (referred to as health intention) were adapted from the scales of Assaker et al. (2011), Prayag et al. (2017), and Xu and Li (2018).
6. The demographic characteristics of the respondents were measured using variables such as gender, age range, education level, income range, and health status.

Considering that the theory of planned behavior is applicable to the prediction of specific behaviors, specific forest health destinations (130–160 km from the place of residence), forest environment pictures, time (at least one night), accommodation prices, forest health curriculum costs, and other related information affecting participation intention were measured by the questionnaire. There are three types of accommodations available: camping with tents (no accommodation), cabins with outdoor bathrooms ($84 per night, with breakfast), and star hotel rooms ($200 per night, with breakfast). The main meal included a nutritious lunch and dinner, and the total cost per capita is between 60 and 90 yuan. There were three types of forest rehabilitation courses: free self-help, 20–60 yuan professional large-scale courses, and 60–100 yuan professional small-scale courses. Most urban residents have the money and time to participate in forest health tourism; that is, the influence of perceived behavioral control on urban residents can be ignored. Therefore, this study measures only the influence of urban residents’ behavioral attitudes and subjective norms on the intention for forest health tourism.

### Research Samples

This study conducted a questionnaire survey of residents in Shenyang from April to June in 2020. Because the participants on Credamo and other survey platforms are mostly highly educated, questionnaire distribution through such a platform will lead to poor representativeness of the sample, so this study used the questionnaire star platform to administer the questionnaire and both online distribution via various WeChat groups of owners in different grades of community and offline distribution. In distributing the questionnaires, four steps were taken to control for common method deviance. (1) Questionnaires were distributed in a unified manner so that the questionnaire issuer would not influence the respondents’ answers or convey individual emotional tendencies to the respondents, emphasizing that there is no right or wrong answer as long as the answer is logical, to ensure the objectivity of the data. (2) Questionnaires were completed anonymously, and the respondents were ensured that their data would be used only for subject studies to reduce concerns and ensure answers were given truthfully. (3) Invalid questionnaires were quickly screened using the reverse-scored items and specified option items. (4) In the process of questionnaire distribution, there were no clear questionnaire quantity requirements to prevent questionnaire distributors from pursuing quantity at the expensive of quality.

Considering that the research objects are urban residents with tourism decision-making power, a screening question, “When you participate in tourism activities (including a day trip), do you usually following relatives and friends (rather than make your own decision)?” was added to the questionnaire, and if the respondent chose “yes,” the answer was deleted. The respondents logged in through WeChat to answer, and each WeChat account and IP address could answer only once to avoid repeated answers. After the questionnaire survey, a total of 465 online questionnaires were submitted, 61 terminated questionnaires were eliminated, and 404 complete questionnaires were received. Then, 183 invalid questionnaires were further eliminated according to the reverse-scored items, the specified items and the filling times, and 221 valid questionnaires were obtained. The recovery rate of valid online questionnaires was only 54.7%. Fifty questionnaires were distributed offline, and 43 valid questionnaires were obtained. The recovery rate of valid offline questionnaires was 86.0%. A total of 264 valid questionnaires were obtained in this survey. Usually, the effective sample size of the structural equation model should be more than ten times the number of observed variables contained in the model (Bentler and Chou, 1987). When the sample size is not less than 200, the model can obtain stable fitting results (Loehlin, 1992). Therefore, it is believed that the number of effective samples obtained in this study can meet the needs of this study. The sample composition is shown in Table 2.

**TABLE 2:** Descriptive statistics of demographic variables.

Variable	Value	Frequency	Percentage(%)	Variable	Value	Frequency	Percentage(%)
Gender	Male	94	35.6	Annual per capita disposable income	Under 20,000	42	15.9
	Female	170	65.4		20,000–50,000	87	33.0
Age	Under 30 years old	36	13.6		50,000–80,000	69	26.1
	30–44 years	100	37.9		80,000–100,000	26	9.8
	45–54 years	68	25.8		100,000–150,000	22	8.3
	55–64 years	43	16.3		150,000–200,000	10	3.8
	Over 64 years old	17	6.4		Over 200,000	8	3.1
Education	Junior high school	20	7.6	Health status	Very healthy	69	26.1
	High school	23	8.7		Healthy	144	54.5
	Junior college	44	16.7		Sub-health	40	15.2
	Undergraduate	118	44.7		Chronic diseases	10	3.8
	Graduate	59	22.3		Severe illness	1	0.4

## Data Analysis and Results

### Common Method Deviation Test

Before the data analysis, it was necessary to detect common method bias, which is frequently observed in questionnaire data. In this study, the Harman single-factor test was used to conduct non-rotational factor analysis on the measurement indicators of all variables in the questionnaire. The results show that the variance interpretation rate of the first common factor obtained by the unrotating factor analysis is 22.509%, which is less than half of the total variance interpretation rate, indicating that the common method deviation is within an acceptable range. Considering that the Harman single-factor test is not sensitive enough, this study uses partial correlation analysis for comparison and verification; that is, the first common factor isolated from the Harman single-factor test is controlled and used to measure the partial correlation between variables, and it is found that no common method bias is obvious. Therefore, it can be considered that there is no common method bias in this study.

### Exploratory Factor Analysis

To ensure that the actual measurement data match the preset conceptual measurement, this study followed three principles for exploratory factor analysis: (1) Each common factor contains at least three measurement items. (2) The non-response probability of measurement items is less than 10%. (3) The measurement items were excluded if the load value in the rotation factor was less than 0.4 or the factor load value was greater than 0.4 for both common factors. Finally, 23 measurement items were retained (see Table 1). Exploratory factor analysis results showed that the KMO value was 0.742, the Bartlett spherical test chi-square value was 2833.996, the df value was 253, and the significance level = 0.000 < 0.05; thus, it is suitable for factor analysis. The characteristic root values of the first six extracted factors were greater than 1, and the total interpretation rate of the cumulative variance in the six factors was 67.027%, which exceeded the extraction limit of 60%, indicating that the extraction of the six factors was reasonable and that the data had good structural validity.

### Reliability and Validity Test

The reliability test is a test method for the reliability analysis of variables in questionnaires. In this study, Cronbach’s α coefficient was used to test the internal consistency of the scale. All Cronbach’s α coefficients are greater than 0.7 (see Table 3), indicating that the variable measurement has good internal consistency and is suitable for further analysis. In this study, combined reliability (CR) and mean variance extraction (AVE) were used to test the convergence and discriminant validity of the research model (see Table 3).

**TABLE 3:** Results of convergent validity analysis.

Variable	Index	Standardized load	Cronbach’s α coefficient	CR	AVE
Frugality	JJ1	0.833	0.792	0.890	0.731
	JJ2	0.764			
	JJ3	0.956			
Cognition	RZ1	0.693	0.811	0.856	0.544
	RZ2	0.706			
	RZ3	0.753			
	RZ4	0.798			
	RZ5	0.734			
Behavioral attitude	TD1	0.711	0.834	0.863	0.557
	TD2	0.741			
	TD3	0.725			
	TD4	0.779			
	TD5	0.774			
Subjective norm	GF1	0.841	0.779	0.861	0.676
	GF2	0.881			
	GF3	0.737			
Health account	ZH1	0.708	0.761	0.840	0.568
	ZH2	0.703			
	ZH3	0.811			
	ZH4	0.787			
Health intention	YX1	0.844	0.901	0.906	0.763
	YX2	0.873			
	YX3	0.903			

The data in Table 3 show that the standardized factor load values of the research model are all greater than 0.7, the AVE values are all greater than 0.5, and the CR values are all greater than 0.8, indicating convergent validity; thus, the research model has good convergent validity.

In the test of discriminant validity (see Table 4), the square root of mean variance extraction (AVE) of all variables in this study was significantly greater than the correlation coefficient between variables, indicating that the data used in this study have good discriminant validity.

**TABLE 4:** Discriminant validity analysis results.

Variable	Frugality	Cognition	Behavioral attitude	Subjective norm	Health account	Health intention
Frugality	0.855					
Cognition	−0.080	0.738				
Behavioral attitude	−0.125	0.403	0.746			
Subjective norm	−0.036	0.093	0.093	0.822		
Health account	−0.172	0.143	0.133	0.121	0.754	
Health intention	−0.070	0.257	0.352	0.302	0.212	0.873

*The value on the diagonal is the square root of the mean variance extraction value (AVE), and the correlation coefficient between variables is below the diagonal.*

### Path Analysis and Hypothesis Testing

In this study, AMOS 24.0 software was used to analyze the overall goodness of fit of the scale data. Among them, χ^2^/DF = 1.518, GFI = 0.905, RMSEA = 0.044, CFI = 0.957, and RMR = 0.047. The absolute fitting index, relative fitting index, and simplified fitting index of the research model reached the ideal level of model fit, indicating that the research model had good fitness.

The path analysis and test results of this study are shown in Table 5.

**TABLE 5:** Path coefficient and test results.

Path	Standardization coefficient	Non-standardized coefficient	SE	CR	*P*
JJ → ZH	−0.152**	–0.084	0.029	–2.917	0.004
JJ → TD	–0.001	–0.001	0.028	–0.034	0.973
RZ → ZH	0.177*	0.145	0.064	2.278	0.023
RZ → TD	0.496***	0.486	0.081	5.982	<0.001
ZH → YX	0.209**	0.252	0.08	3.143	0.002
GF → YX	0.282***	0.293	0.069	4.235	<0.001
TD → YX	0.281***	0.284	0.078	3.650	<0.001
JJ → YX	–0.040	–0.027	0.025	–1.089	0.276
RZ → YX	0.095	0.094	0.071	1.312	0.190

*p < 0.05. **p < 0.01. ***p < 0.001.

According to the results in Table 5, the standardized path coefficient between frugality and health accounts (JJ → ZH) is −0.152 (*P* < 0.01), indicating that frugality significantly negatively affects health accounts; thus, H1 is verified. The standardized path coefficient between frugality and behavioral attitude (JJ → TD) is −0.001 (*P* > 0.05), indicating that frugality does not significantly affect the attitude of urban residents toward forest health tourism, so H2 is not verified. For urban residents, frugality affects the establishment of their health accounts. However, frugality is aimed at reducing unnecessary costs. When urban residents realize that forest health tourism has a good health care effect, their attitude is not affected by frugality.

The standardized path coefficient between cognition and health accounts (RZ → ZH) was 0.177 (*P* < 0.05), indicating that urban residents’ cognition of forest health tourism had a significant positive effect on health accounts, so H3 is verified. The standardized path coefficient between cognition and behavioral attitude (RZ → TD) was 0.496 (*P* < 0.001), indicating that urban residents’ cognition of forest health tourism had a significant positive effect on their attitude toward forest health tourism, so H4 is verified. Urban residents’ awareness of forest health has a significant effect on their health accounts and forest health tourism attitudes.

The standardized path coefficients of health accounts, subjective norms, behavioral attitudes, and forest health tourism intentions are 0.209 (*P* < 0.05), 0.282 (*P* < 0.001), and 0.281 (*P* < 0.001), respectively, indicating that health accounts, subjective norms, and behavioral attitudes have significantly positive effects on the forest health tourism intentions of urban residents, verifying H5, H6, and H7. The standardized path coefficients between frugality and forest health tourism intention (JJ → YX) and between cognition and forest health tourism intention (RZ → YX) are 0.04 (*P* > 0.05) and 0.095 (*P* > 0.05), respectively, indicating that the effect of frugality and cognition on forest health tourism intention is not significant, and a further mediating effect test is needed.

### Test of Mediating Effect

In this study, the bootstrap method was used to test the mediating effect of frugality on the relationship between cognition and forest health tourism intention. The bootstrap method determines the significance of the mediating effect of the model by whether there is 0 between the upper and lower critical values. The specific mediating effect test results are shown in Table 6.

**TABLE 6:** Standardized bootstrap mediation effect test.

Path	Estimate	SE	Bias-corrected 95%CI			Percentile 95%CI		
			Lower	Upper	*P*	Lower	Upper	*P*
Stdind JJ1	−0.032	0.015	−0.077	−0.011	0.001	−0.065	−0.007	0.003
Stdind JJ2	0.000	0.016	−0.029	0.034	0.934	−0.028	0.036	0.975
Stdind RZ1	0.139	0.046	0.07	0.257	0.001	0.064	0.242	0.001
Stdind RZ2	0.037	0.020	0.007	0.090	0.011	0.002	0.078	0.030

*JJ1 indicates: frugality → health account → forest health tourism intention. JJ2 indicates frugality → behavioral attitude → forest health tourism intention. RZ1 indicates cognition → behavioral attitude → forest health tourism intention. RZ2 indicates cognition → health account → forest health tourism intention.*

**FIGURE 2:**
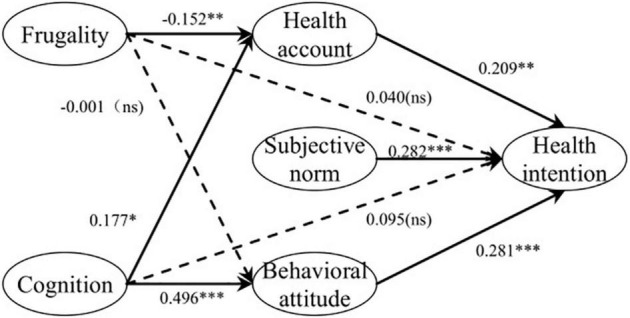
Hypothesis test results. *p < 0.05. ^**^p < 0.01. ^***^p < 0.001. ns represents *p* is not significant. The unbroken line represents significant paths, while the dotted line represents non-significant paths.

The *P* value and confidence interval in Table 6 show that the JJ2 path is not significant; that is, behavioral attitude does not have a significant mediating effect on the relationship between frugality and forest health tourism intention, so H9a is not verified. The other three paths are significant; that is, the health account has a significant full mediating effect on the relationship between frugality and forest health tourism intention, verifying H8a. At the same time, health accounts and behavioral attitudes have a significant full mediating effect on the relationship between cognition and intention to engage in forest health tourism, verifying H8b and H9b. The path coefficients between variables after the hypothesis test are shown in Figure 2.

## The Mediating Effect Analysis Based on Multigroup Comparison

### Mediating Effect Analysis Based on Latent Variable Grouping

The above path analysis shows that frugality and cognition have significant effects on health accounts. Subjective norms and behavioral attitudes have significant effects on forest health tourism intention. Health accounts have a significant full mediating effect on the relationship between frugality and forest health tourism intention. To further test whether cognition, subjective norms and behavioral attitudes have a moderating effect on the path of frugality → health account → forest health intention, AMOS24.0 was used for multigroup analysis of the questionnaire data. Therefore, the variables of cognition, subjective norms, and behavioral attitude were transformed into binary variables. Taking cognition as an example, it is necessary to first generate a variable of cognitive mean value and assign it to the mean value of each cognitive measurement item. Then, the cognitive mean of all samples is calculated. Finally, the cognitive variables above the mean value are assigned a value of 1, indicating the high-cognitive group, and the cognitive variables below the mean value are assigned a value of 0, indicating the low-cognitive group. Similarly, according to subjective norm assignment, the samples are divided into a high norm group and a low norm group, and according to behavioral attitude assignment, the samples are divided into a positive attitude group and a negative attitude group.

Multigroup analysis showed that the CFI, TLI, and IFI values in the model were between 0.9 and 0.96, higher than the standard value of 0.9. The RMSEA values were between 0.05 and 0.06, lower than the critical value of 0.08. This shows that the multigroup analysis model has a good adaptation effect with the sample data. The results of the multigroup path analysis are shown in Table 7.

**TABLE 7:** Results of multigroup analysis based on cognition, subjective norms, and behavioral attitudes.

Hypothesis path	Public cognition		Subjective norm		Behavioral attitude	
	Low cognition	High cognition	Low norm	High cognition	Negative attitude	Positive attitude
H1: JJ → ZH	−0.154**	–0.052	−0.148**	–0.031	−0.108**	–0.058
H5: ZH → YX	0.245*	0.308**	0.242*	0.446**	0.305*	0.317**
H8a: JJ → YX	0.015	0.024	0.026	0.020	0.007	0.017

*P < 0.05, **P < 0.01.

Table 7 shows that in the influence path H1 of frugality on health accounts, the influence on low-cognitive people is significant (β = −0.154, *P* < 0.01), but the effect on high-cognitive people is not (β = −0.052, *p* > 0.05), indicating that in the process of setting up health consumption mental accounts, the higher the urban residents’ awareness of forest health is, the higher the perceived value of consumption is; thus, the inhibitory effect of frugality on health consumption mental accounts is not significant. In terms of subjective norms, the frugality habits of the low-cognitive group still significantly inhibited the mental account of health consumption (β = −0.148, *P* < 0.01), while no significant effect of frugality occurred in the high-cognitive group (β = −0.031, *p* > 0.05), indicating that the inhibitory effect of frugality on the mental account of health consumption is no longer significant with strong herd mentality and group consciousness. The frugality habits of urban residents who hold negative attitudes toward forest health still significantly inhibit mental health consumption accounts (β = −0.108, *P* < 0.01); however, for the urban residents with a positive attitude toward forest health, their frugal habits had no significant inhibitory effect on the mental account of health consumption (β = −0.058, *P* > 0.05).

In path H5 of the effect of health accounts on the intention for forest health tourism, there is no significant difference across groups, indicating that the positive effect of health accounts on the intention for urban residents to engage in forest health tourism is stable and is not related to the level of cognition or subjective norms or whether the attitude is positive or negative.

In the influence path H8a of frugality on urban residents’ intention to engage in forest health tourism, there was no significant difference between groups, indicating that frugality affects the intention to engage in forest health tourism only through health accounts.

In summary, for urban residents with low awareness of forest health, low levels of subjective norms and negative attitudes, the complete mediating effect of health accounts on the relationship between frugality and forest health tourism intention always exists. That is, for this group, frugality inhibits the establishment of health accounts, which has a significant negative effect on forest health tourism intention.

### Mediating Effect Analysis Based on Demographic Variables Grouping

To explore the differences in path parameters between frugality → health account → forest health intention across groups, four demographic variables, gender, education, age, and income, were selected for multigroup analysis. For education, a bachelor’s degree was used as the boundary, with those with less than a bachelor’s degree in the low-degree group and those with a bachelor’s degree or above in the high-degree group. In terms of age, referring to the WHO’s standard of dividing the boundaries between young people and middle-aged people at 45 years of age, the group under 45 years of age was classified as the young group, and the group over 45 years old was classified as the middle-aged group. In terms of income, the Shenyang per capita annual disposable income of 50,000 yuan was used as the boundary, with those earning below 50,000 yuan classified into the low-income group and those earning 50,000 yuan or above classified into the high-income group. The CFI, TLI, and IFI values in the multigroup analysis model were between 0.9 and 0.95, higher than the standard value of 0.9. The RMSEA values were between 0.034 and 0.046, lower than the optimal threshold of 0.05. This shows that the multigroup analysis model has a good adaptation effect with the sample data. This study focuses on hypotheses H1, H5, and H8a for multigroup analysis (see Table 8).

**TABLE 8:** Results of multigroup analysis based on demographic variables.

Hypothesis path	Gender		Education		Age		Income	
	Male	Female	Low education	High education	Youth	Middle-aged	Low income	High income
H1: JJ → ZH	–0.058	−0.086**	−0.138*	–0.008	−0.122**	–0.046	−0.097*	–0.015
H5: ZH → YX	0.162	0.303**	0.282*	0.212*	0.239	0.189*	0.409**	0.139
H8a: JJ → YX	0.014	0.035	0.041	0.016	0.007	0.052	0.026	0.005

*P < 0.05, **P < 0.01.

Table 8 shows that in path H1 of the effect of frugality on health accounts, the effect on women is significant (β = −0.086, *P* < 0.01), while there is no significant effect on men (β = −0.058, *P* > 0.05), indicating that the more frugality women show, the lower their health accounts. In other words, women’s frugal habits have a significant inhibitory effect on their mental accounts of health consumption, but there is no significant inhibitory effect on men. In terms of education, the effect on low-educated people is significant (β = −0.138, *P* < 0.05) but not on those with higher education (β = −0.008, *P* > 0.05), indicating that highly educated people are more clearly aware of the role of forest health than less-educated people. Even if they are very frugal, they will set up health accounts, so the effect is not significant. In terms of age, the effect on young people was significant (β = −0.122, *P* < 0.05), but that on middle-aged people was not (β = −0.046, *P* > 0.05), indicating that frugal middle-aged and elderly people will still set up higher health accounts. In terms of income, the effect on low-income people was significant (β = −0.097, *P* < 0.05), but that on high-income people was not (β = −0.015, *P* > 0.05), indicating that frugal high-income and low-income people set up higher health accounts.

In path H5 of the effect of health accounts on the intention for forest health tourism, the effect is significant for women (β = 0.303, *P* < 0.01) but not for men (β = 0.162, *P* > 0.05), indicating that women are more likely to plan consumption expenditures. Once the health account is set low, the intention for forest health tourism is also low. In terms of education, the effect is significant for both the low-educated (β = 0.282, *P* < 0.05) or high-educated (β = 0.212, *P* < 0.05) groups. In terms of age, there was a significant effect on the elderly (β = 0.189, *P* < 0.05) but not on the young (β = 0.239, *P* > 0.05), indicating that the higher the health accounts established by the elderly are, the stronger their intention for forest health tourism. In terms of income, the effect on low-income people is significant (β = 0.409, *P* < 0.01), but that on high-income people is not (β = 0.139, *P* > 0.05), indicating that the lower the health account of low-income people is, the weaker the intention for forest health tourism. Even if the health account of high-income people is set very low, there is no significant effect on their intention for forest health tourism.

In the influence path H8a of frugality on urban residents’ intention to engage in forest health tourism, there was no significant difference between groups, indicating that frugality affects the intention for forest health tourism only through the health accounts for different types of urban residents.

## Research Conclusion and Discussion

### Research Conclusion

Based on the theory of planned behavior, mental account theory, and the cognition-attitude-behavior model, this study explored the mechanism by which frugality and cognition affect the intention for forest health tourism, in which health accounts have a complete mediating effect on the relationship between frugality and the intention for forest health tourism, and analyzed the differences of mediating effect of the path “frugality—health accounts—intention” through the grouping of cognition, subjective norms, behavioral attitude, and demographic variables. The conclusions were as follows:

1. Health accounts fully mediate the relationship between frugality and forest health tourism intention, and frugality has a significant negative effect on health accounts, thereby inhibiting forest health tourism intention. However, for urban residents who agree with the role of forest health and hold a positive attitude or are vulnerable to the influence of surrounding relatives and friends, the inhibition of frugality is longer significant.
2. Health accounts and behavioral attitudes play a full mediating role in the relationship between cognition and forest health tourism intention. The higher the urban residents’ awareness of the role of forest health are, the higher their health accounts and the stronger their intention to participate in forest health tourism. Health accounts, behavioral attitudes and subjective norms have positive effects on urban residents’ intention to engage in forest health tourism; that is, cognition has an indirect positive effect on urban residents’ intention to engage in forest health tourism through health accounts and behavioral attitudes.
3. Health accounts have a fully mediating effect on the relationship between frugality and forest health tourism intention, and this effect is moderated by demographic variables such as gender, education, age, and income. The inhibitory effect of frugality on the intention for forest health tourism is no longer significant among male, highly educated, elderly, and high-income urban residents.

### Theoretical Contribution

Firstly, this research expanded the application of mental account theory in the study of forest health tourism intention of urban residents. In the previous study of tourists’ forest health tourism intention, the theory of planned behavior was singly used to explore the influence mechanism of behavioral intention (Xie et al., 2019), which was relatively weak in explanation. Introducing the concept of frugality and combining the theory of planned behavior with mental account theory, this study analyzed the effect mechanism of frugality and cognition on the forest health tourism intention of urban residents, which not only broadened the application boundary of mental account theory, but also helped to enrich the theoretical system of tourism consumption behavior.

Secondly, in the past, the research on tourism behavioral intention based on the theory of planned behavior (including the extended theory of planned behavior) usually did not provide specific information such as tourist destination and price that affected consumption attitude in the questionnaire explanation, which made it difficult to measure the real consumption attitude and specific behavior intention. On the premise of clearly defining the specific behavior of forest health tourism, this study verified the effect of cognition, behavioral attitude and subjective norms on forest health tourism intention, and further took them as moderating variables to expand the relationship between related concepts of the theory of planned behavior and enhance the comprehensive explanatory power of the theory.

### Management Enlightenment

This study introduces the concept of frugality, combines the theory of planned behavior with the theory of mental accounts, and analyses the mechanism by which frugality and cognition affect urban residents’ intention to engage in forest health tourism. It not only broadens the application boundary of mental account theory and expands the relationship between related concepts of planned behavior theory but also enriches and improves the theoretical system of tourism consumption behavior. To alleviate the inhibitory effect of frugality, strengthen urban residents’ awareness of forest health tourism and promote the development of the forest health industry, this study makes three suggestions:

1. The forest health base, through the continuous enrichment of marketing and publicity, aims to improve the visibility of forest health tourism destinations and strengthen forest health cognition and consumption habits. Forest health tourism is new and has not been widely recognized, and urban residents do not understand its advantages in disease prevention and health care. Therefore, strengthening forest health tourism marketing propaganda is imperative. On the one hand, the forest health base can realize the continuous push of forest health tourism-related information and raise public awareness through various social media (WeChat, Microblog, etc.), short video new media (Tik Tok, Kwai, etc.) and even search engine platforms (Baidu, Google, etc.). On the other hand, middle-aged and elderly people are the main consumers in the forest health tourism market, and their ability to obtain information related to forest health tourism is weak. Most elderly people are still used to traditional media, so it is necessary to increase the propaganda of traditional media such as television advertising and guide young people to buy forest health product packages for their parents from the perspective of children’s filial piety to their parents to cultivate middle-aged and elderly people’s awareness of forest health and consumption habits.
2. Because frugal consumers are more inclined to make rational use of their money to maximize revenue, forest health tourism bases provide free forest health projects with small investments to enhance the experience value of tourists. For example, only yoga mats and video playback devices are needed for open forest yoga and meditation courses, which will greatly improve consumers’ perceived benefits and play an important role in fostering positive attitudes toward forest health tourism and mitigating the negative effect of frugality. At the same time, in the early stage of promotion, the forest health tourism base should increase the benefits of group health and package meals and provide forwarding and recommendation point exchange activities, which can not only increase household consumption but also drive existing consumers to recruit potential consumers via word-of-mouth.
3. Through market segmentation, forest health tourism bases select the target market that matches the supply capacity of enterprises and formulate targeted marketing strategies. The results of multigroup analysis based on demography showed that high education, high income, and middle-aged and elderly male residents were less affected by frugality in the establishment of health accounts. Therefore, the forest health tourism base should target highly educated, high-income, and middle-aged and elderly consumer groups (mainly males) via advertising. First, it should provide a package of forest health tourism products for family consumption by taking the consumption concept of high life quality, high cost-performance ratio, and natural health care over treatment as the starting point and drive the demand for forest health tourism among the relatives and friends of experienced tourists. Second, the forest health base should provide various levels of forest health tourism products and services, including offering fee-based professional courses and free self-help courses for Tai Chi, yoga and meditation enthusiasts to meet the needs of urban residents at various levels of demand.

This study is restricted by many factors and has certain limitations, mainly including the following: (1) To ensure the quality of the questionnaire, this study strictly controlled the quality of questionnaire responses, resulting in a limited number of valid questionnaires. The relevant research conclusions need to be further verified by collecting large sample data from multiple regions. (2) Using a multigroup analysis method, latent variables such as cognition, subjective norms and behavioral attitudes are treated as significant variables, ignoring the measurement error of variables, which may cause an underestimation of the mediation and moderating effects. (3) Since the forest health industry is in the early stage of development, it is difficult to collect relevant data on forest health tourism behavior. This study analyses only the behavioral intention level. In the future, field experimental research methods will be used to study the marketing effect of forest health combined products.

## Data Availability Statement

The original contributions presented in the study are included in the article, further inquiries can be directed to the corresponding author.

## Ethics Statement

Ethical review and approval was not required for the study on human participants in accordance with the local legislation and institutional requirements. Written informed consent for participation was not required for this study in accordance with the national legislation and the institutional requirements.

## Author Contributions

YL contributed to the conception of the study and helped perform the analysis with constructive discussions. QH performed the experiment, data analysis, and wrote the manuscript. YL and TW contributed significantly to analysis and manuscript preparation. All authors contributed to the article and approved the submitted version.

## Conflict of Interest

The authors declare that the research was conducted in the absence of any commercial or financial relationships that could be construed as a potential conflict of interest.

## Publisher’s Note

All claims expressed in this article are solely those of the authors and do not necessarily represent those of their affiliated organizations, or those of the publisher, the editors and the reviewers. Any product that may be evaluated in this article, or claim that may be made by its manufacturer, is not guaranteed or endorsed by the publisher.

## Footnotes

1 http://www.gov.cn/zhengce/content/2019-07/15/content_5409492.htm, Opinions of the State Council on Implementing Healthy China Action (National Development (2019)13).
